# Novel Mouse Models of Fungal Asthma

**DOI:** 10.3389/fcimb.2021.683194

**Published:** 2021-08-16

**Authors:** Michael Daines, Rhea Pereira, Aubrey Cunningham, Barry Pryor, David G. Besselsen, Yuchen Liu, Qianwen Luo, Yin Chen

**Affiliations:** ^1^Department of Pediatrics, College of Medicine, University of Arizona, Tucson, AZ, United States; ^2^Asthma & Airway Disease Research Center, University of Arizona, Tucson, AZ, United States; ^3^School of Plant Science, University of Arizona, Tucson, AZ, United States; ^4^Animal and Comparative Biomedical Sciences, University of Arizona, Tucson, AZ, United States; ^5^Department of Pharmacology and Toxicology, School of Pharmacy, University of Arizona, Tucson, AZ, United States; ^6^Department of Biosystems Engineering, University of Arizona, Tucson, AZ, United States

**Keywords:** *Alternaria*, asthma, AHR, lung, fungus, pathophysiology

## Abstract

*Alternaria alternata* is a ubiquitous fungus and a major allergen associated with the development of asthma. Inhalation of intact spores is the primary cause of human exposure to fungal allergen. However, allergen-rich cultured fungal filtrates are oftentimes used in the current models of fungal sensitization that do not fully reflect real-life exposures. Thus, establishing novel spore exposure models is imperative. In this study, we established novel fungal exposure models of both adult and neonate to live spores. We examined pathophysiological changes in the spore models as compared to the non-exposure controls and also to the conventional filtrate models. While both *Alternaria* filtrate- and spore-exposed adult BALB/c mice developed elevated airway hyperresponsiveness (AHR), filtrates induced a greater IgE mediated response and higher broncholavage eosinophils than spores. In contrast, the mice exposed to *Alternaria* spores had higher numbers of neutrophils. Both exposures induced comparable levels of lung tissue inflammation and mucous cell metaplasia (MCM). In the neonatal model, exposure to *Alternaria* spores resulted in a significant increase of AHR in both adult and neonatal mice. Increased levels of IgE in both neonatal and adult mice exposed to spores was associated with increased eosinophilia in the treatment groups. Adult demonstrated increased numbers of lymphocytes that was paralleled by increased IgG1 production. Both adults and neonates demonstrated similarly increased eosinophilia, IgE, tissue inflammation and MCM.

## Introduction

The prevalence of asthma has significantly increased in United States and in other industrialized countries (Sunyer et al., 1999). The prelude of asthma development is usually a repeated environmental allergen exposure and sensitization leading to type 2 immune response (or T2IR) (Nelson et al., 1999; Busse and Mitchell, 2007). T2IR refers to both innate and adaptive arms of the immunity that is developed mainly for the defense against parasitic infection, and characterized by participating cells (e.g. CD4+ T_H_2 cells, ILC2s, eosinophils, basophils, mast cells, alternatively activated macrophages), antibodies (e.g., the IgE antibody subclass) and cytokines (e.g. IL-4, IL-13, thymic stromal lymphopoietin (TSLP), IL-25 and IL-33) (Wynn, 2015). Asthma is a T2IR-mediated disease characterized by lower airway chronic inflammation, MCM, and AHR (Lambrecht and Hammad, 2015).

Fungal exposure has long been recognized as a significant risk factor for asthma. Being the major components of indoor molds, fungal sensitization during the first 2 years of life was found to be associated with an increased risk of developing asthma in the meta-analysis of 8 birth cohorts in Europe. (Tischer et al., 2011) The prevalence of fungal sensitization in general asthmatics is 28% on average (as high as 48%) (Agarwal, 2011). Fungal asthma is oftentimes poorly managed with frequent exacerbations and hospitalizations (Denning et al., 2006; Denning et al., 2014; Sharpe et al., 2015; Masaki et al., 2017). “Severe Asthma with Fungal Sensitization” or SAFS has been coined for a type of severe asthma with the sensitization to *Alternaria, Aspergillus, Cladosporium or Penicillium* (Denning et al., 2006).

Studies from our and other groups have demonstrated that *Alternaria* sensitization was associated with asthma development (Peat et al., 1993; Halonen et al., 1997; Katz et al., 1999; Dowaisan et al., 2007; Ezeamuzie et al., 2000; Salo et al., 2006), and sometimes severe or even fatal asthma (Downs et al., 2001; Bush and Prochnau, 2004). Furthermore, efforts to reduce indoor *Alternaria* exposure by extensive cleaning have been proven impossible as demonstrated in the HEAL study (Grimsley et al., 2012). In addition to the classical CD4+ T-helper 2 (T_H_2) cells in *Alternaria* sensitization, *Alt* filtrate, the fungal secretome, was reported to induce ILC2 expansion, subsequent production of IL-5/IL-13 and eosinophilia, depending on IL-33-ST2 signaling (Bartemes et al., 2012). Additionally, acute *Alternaria* exposure can cause asthma exacerbation, independent of *Alternaria* sensitization (Tham et al., 2017). Because of its strong link to asthmagenic T2IRs at both innate and adaptive arms, *Alternaria* exposure can be a good surrogate model to study fungal asthma.

Fungal filtrate extract or filtrate model is to expose mice with allergen-rich fungal secretions, and it is a robust allergen model that has been widely used. However, the effect caused by intact fungal spore, a common human exposure to fungal species (Baxi et al., 2016), is lacking in this model. An average person exposing to a large number of fungal spores each day, up to 50,000 spores per cubic meter of air during the fungal season (Pashley et al., 2012). The previous attempt to establish a fungal asthma model using *Alternaria* spore inhalation alone has failed because of cachexia (Denis et al., 2007). Thus, to study this important fungal allergen, we seek to establish a fungal asthma model by exposing mice to *Alternaria* spores *via* an inhalational route.

## Methods

### Fungal Spores and Filtrates

*Alternaria alternata* (ATCC 66981) spores were produced on V8 agar (V8A: 200 ml V8 juice, 2 g CaCO3, 15 g agar in one liter H20) incubated at room temperature for a week under regular room lighting (12/12 hrs light/dark). Spores were collected in HBSS by gentle agitation and quantified on a hemacytometer. The Lyophilized mass of *Alternaria* filtrates (GREER, Lenoir, NC) was dissolved in HBSS (Hank’s Balanced Salt Solution) to make a 100X stock solution.

### Mice Exposures

The animal protocol was approved by the Institutional Animal Care and Use Committee (IACUC) at the University of Arizona. 5-week old adult BALB/c mice were briefly anesthetized with 3% isofluorane and intranasally administered with *Alternaria* spores (10^5^) in a 100 µl HBSS, 10 µg filtrates or the same volume of HBSS (the solvent of the spores or filtrates) weekly for total 6 weeks. For the experiments with neonates, 5-week old adults and 1-week neonatal BALB/c mice were placed under isoflurane and intranasally administered *Alternaria* spores or HBSS weekly for a period of 6 weeks. The adult dose was kept constant as described above. The pups were weighed and were given a weight-proportioned reduced dose and volume. 8-10 animals were used for each group.

### Airway Hyperresponsiveness (AHR)

Mice were inhalationally challenged with different doses of methacholine. AHR was measured by the FlexiVent^®^ system (SCIREQ, Montreal, Canada). Peak resistance values at each dose were plotted against the corresponding methacholine dose.

### Bronchoalveolar Lavage (BAL), Differential Cell Count and Cytokine Measurement

Lungs were instilled twice with 1mL HBSS to collect BAL. The cells in the BAL were cytocentrifuged, air-dried, stained with HEMA 3 stain set (Thermo Scientific, Gilbert, AZ) and the number of macrophages, neutrophils, eosinophils, and lymphocytes were then counted in a blinded manner using light microscopy by at least two researchers to ensure an objective evaluation. Differential cell counts (macrophage, neutrophil, eosinophil, lymphocyte) were presented as the number of each cell type. IL-13 or IL-17 in the BAL was measured by ELISA kits from R&D Systems (Minneapolis, MN).

### Serology

Blood was collected from the femoral artery and allowed to clot at ambient temperature (19-24°C). Serum was separated by centrifugation and total immunoglobins (IgE, IgG1) were measuring using ELISA kits (BD Pharmingen, CA) according to manufacturers instructions.

### Lung Histology

Lungs were fixed, sectioned and stained at the Pathology Services Laboratory, University Animal Care. Slides were stained for inflammatory cells (H&E stain) and goblet cells (PAS stain). Pathology scores and analysis were performed by Dr. Besselsen (DVM, PhD) at the University animal service core.

### Statistical Analysis

All values are given as the Means ± SEM. Statistics were evaluated with GraphPad software (San Diego, CA). Multivariate comparisons were made using ANOVA with Bonferroni’s multiple-comparison posttest. Differences with p<0.05 were considered statistically significant.

## Results

### An Adult Model of Asthma With Fungal Sensitization

**Figure 1:**
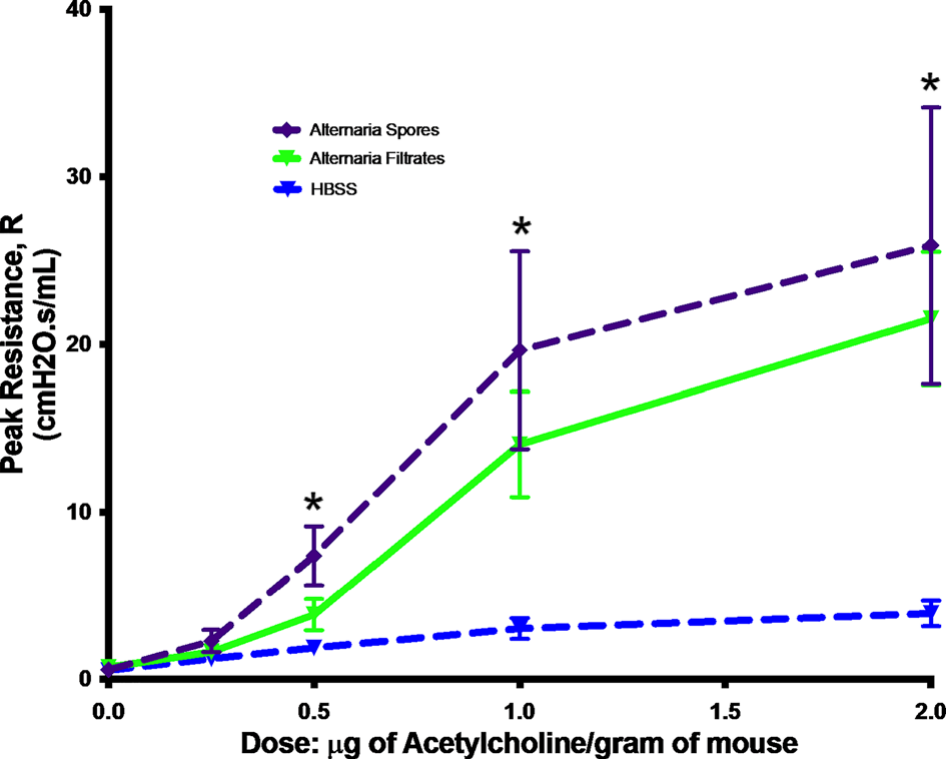
*Alternaria*-induced AHR. Mice were exposed to *Alternaria* filtrates, spores, or HBSS controls. Peak resistance after methacholine challenge was presented as Mean ± SEM, n = 8/group. *P < 0.05.

To mimic a real-life human exposure, we exposed mice with live spores of *Alternaria*. We compared this new model with the commonly used filtrate model. As asthma is a chronic disease, we opted for the long-term exposure. Additionally, we relied entirely on airway exposure to spores or filtrates as this is the dominant route of exposure in a real life. In a previous study, chronic instillation of *Alternaria* spores was found to induce cachexia, therefore was stopped after 5 weeks (Denis et al., 2007). In the present study, we tried 6-week exposure and found no weight loss or other outright morbidity (data not shown). Instead, AHR, a hallmark of asthma, was elevated in mice exposed to both *Alternaria* filtrate and spores when compared to controls. Interestingly, the differences between filtrate and spore model were not statistically significant (**Figure 1**), suggesting our spore model demonstrated same level of AHR increase as the commonly used filtrate model.

**Figure 2:**
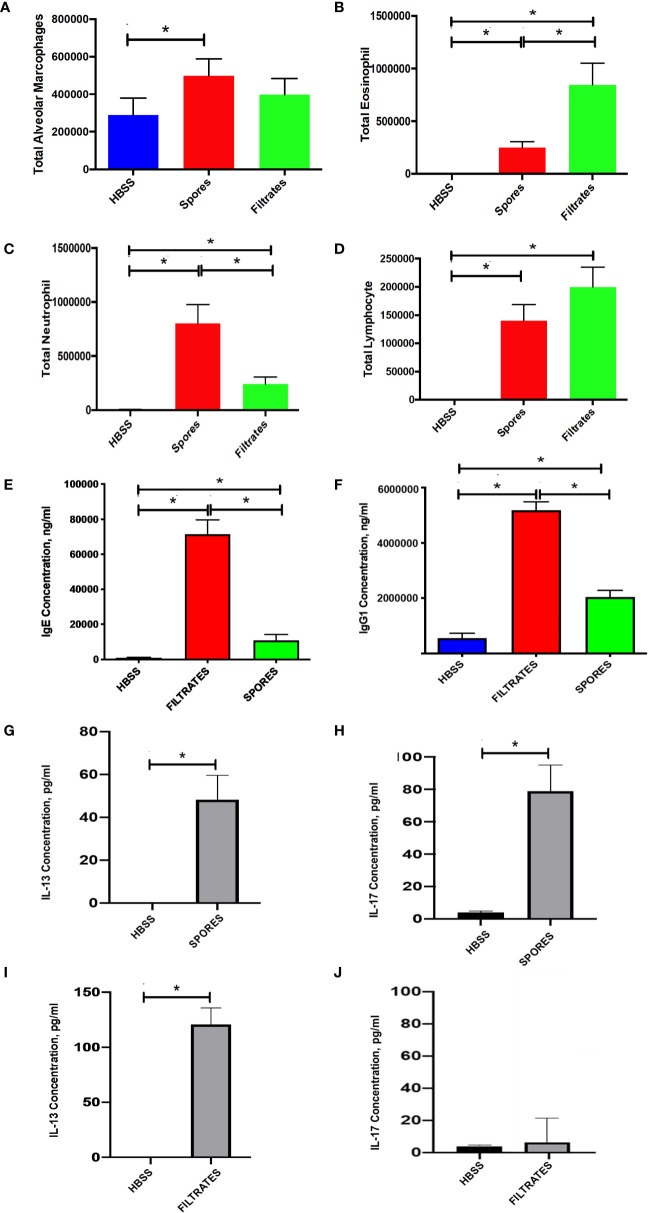
BAL cell counts and serology in the filtrate or spore model. Data represents mean ± SEM, n = 8-10/group. *P < 0.05. **(A–D)** Differential cell counts. Different cell types in BAL samples were counted from these mice and presented as the total cell number. **(E, F)** Serological testing for total serum IgE and IgG1. **(G, H)** BAL IL-13 and IL-17 in the mice treated with spores. **(I, J)** BAL IL-13 and IL-17 in the mice treated with filtrates.

Then, we wanted to investigate other pathophysiological changes in the spore model as compared to the filtrate model. By a differential cell count on BAL, we found that lung inflammation was increased in mice exposed to either spores or filtrates but the type of inflammatory response was different. Spore exposure moderately enhanced macrophage number, but filtrate did not (**Figure 2A**). Spore exposure resulted in a greater neutrophilic inflammation (**Figure 2C**), while filtrate exposure caused a higher eosinophilic (**Figure 2B**) and lymphocytic inflammatory response (**Figure 2D**). Thus, despite the observation that both exposures induced comparable AHR, underlying inflammatory mechanisms were likely different. Nonetheless, both models demonstrated eosinophilic inflammation indicating an asthma related T_H_2 response. Furthermore, we tested serum antibody responses. Indeed, serum IgE was significantly increased in both models, and the filtrate model had a much greater IgE response as compared to the spore model (**Figure 2E**). This observation was consistent with the BAL eosinophil counts. Thus, the filtrate exposure appeared to induce a much stronger T_H_2 response than the spore exposure. We also tested IgG response, a T_H_1 marker for an antifungal defense. IgG1 was elevated in both models, and again filtrates induced higher IgG1 than spores (**Figure 2F**). But the magnitude of difference for IgG1 (~2.5 fold) was not as great as IgE (~6.8 fold). Interestingly, while spore exposure induced a strong production of both IL-13 (**Figure 2G**) and IL-17 (**Figure 2H**) in the BAL, filtrates treatment only induced IL-13 (**Figure 2I**), but not IL-17 (**Figure 2J**). Thus, although the filtrate exposure may induce a strong T_H_1 and T_H_2 immunological responses, only spores could induce a robust T_H_17 response.

**Figure 3:**
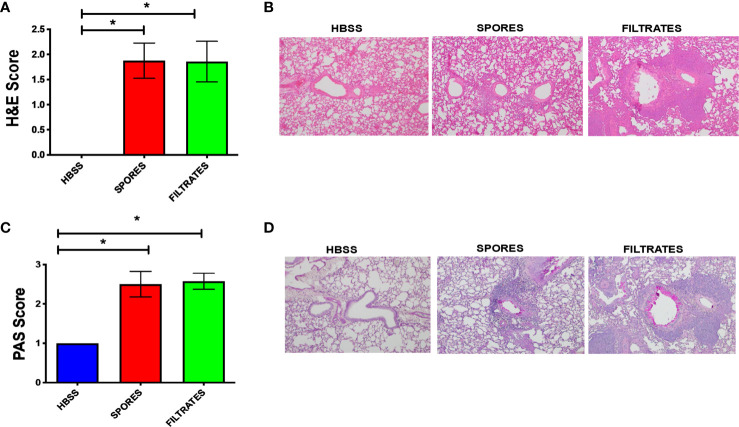
Histological analyses of the filtrate or spore model. *p < 0.05, n = 8. **(A)** H&E scores. **(B)** Example H&E images. **(C)** PAS scores. **(D)** Example PAS images.

We further examined the tissue histology in these two models. H&E scores (**Figure 3A**) and example figures (**Figure 3B**), indicating tissue inflammation, were identical in both models, suggesting a similar level of lung inflammation despite the difference of infiltrated inflammatory cells (**Figure 2**). Mucous cell metaplasia (MCM), another hallmark of asthma indicating lung remodeling, was measured by a PAS staining. The level of MCM was identical following exposure to filtrates or spores (**Figures 3C, D**).

### An Asthma Model of Early-Life Fungal Exposure

**Figure 4:**
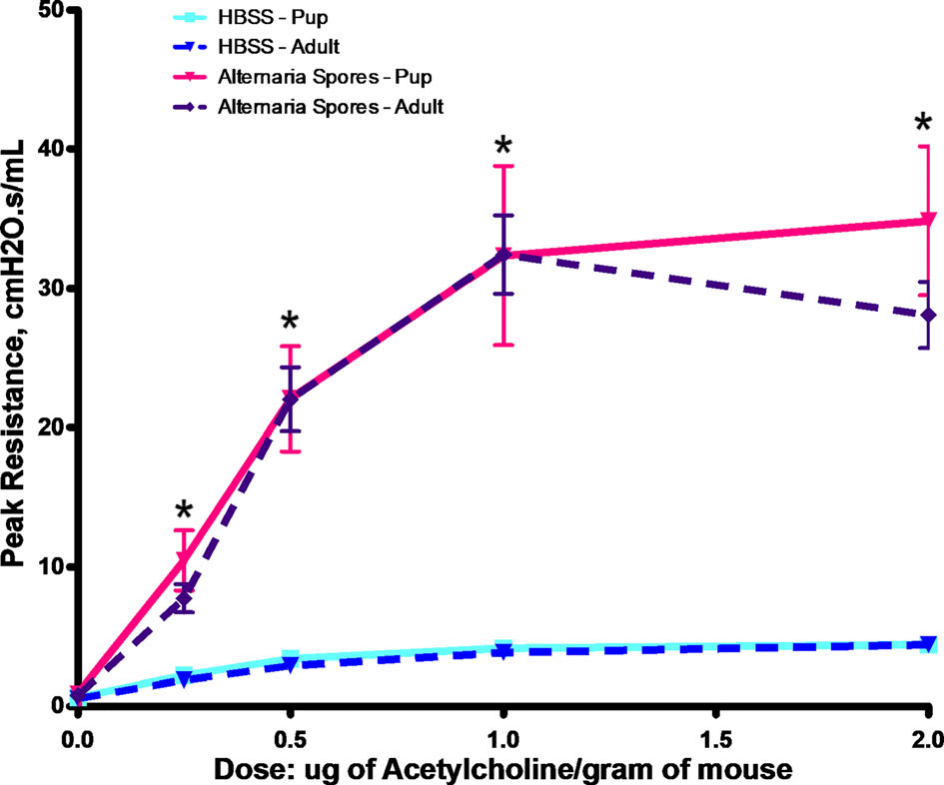
*Alternaria* spore-induced AHR in adults or neonates. Adult mice or neonates were exposed to spores or HBSS controls. Peak resistance after methacholine challenge was presented as Mean ± SEM, n = 8/group. *P < 0.05.

Childhood asthma can be difficult to treat, and it is also strongly associated with sensitization to the *Alternaria*. Thus, we decided to develop a mouse model with exposures starting at 7 days of age. As shown in **Figure 4**, exposure to *Alternaria* spores resulted in a significant increase of AHR in both adult and neonatal mice throughout the acetylcholine dose range. However, there was no significant difference between spore treated adults and neonates (**Figure 4**).

**Figure 5:**
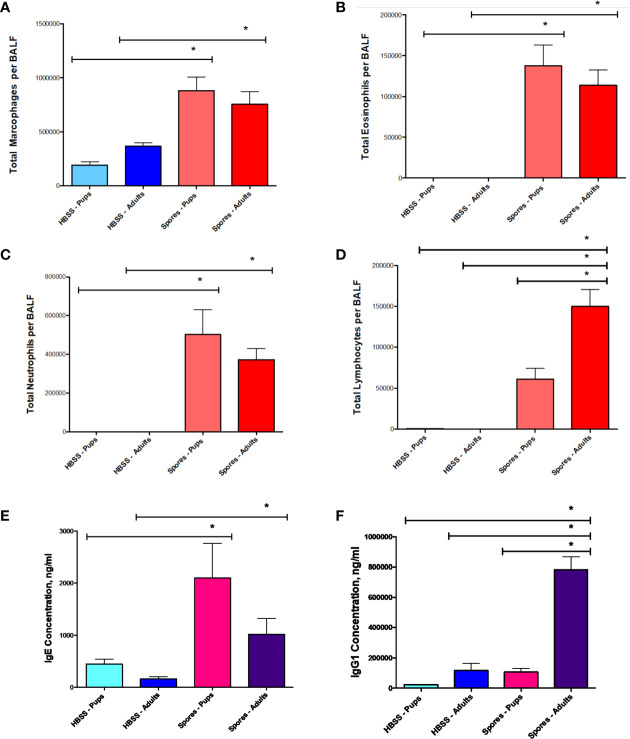
BAL cell counts and serology in adults or neonates. Data represents mean ± SEM, n = 8-10/group. *P < 0.05. **(A–D)** Differential cell counts. Different cell types in BAL samples were counted from these mice and presented as the total cells. **(E, F)** Serological testing for total serum IgE and IgG1.

Then, we examined airway inflammatory responses by a differential cell count. Spore exposure induced a moderate elevation of macrophages (**Figure 5A**), but a significant increase of both neutrophils (**Figure 5C**) and eosinophils (**Figure 5B**) in both adults and neonates at almost identical magnitudes. Interestingly, adults had more lymphocytes than neonates in BAL (**Figure 5D**). We further tested serum antibody responses. The increased levels of IgE were observed in both neonatal and adult mice exposed to spores (**Figure 5E**). IgE in neonates had a trend of increase as compared with adults, but it was not statistically significant. In contrast, serum IgG1 in adults was much higher than in neonates (**Figure 5F**), which was paralleled with an increase in lymphocytes in adults (**Figure 5D**). This observation is consistent with the notion that adults usually generate a stronger IgG based immunity than neonates (Adkins et al., 2004; Levy, 2007). Interestingly, IgE responses in both groups appeared to be similar, suggesting that both can develop comparable allergic responses.

**Figure 6:**
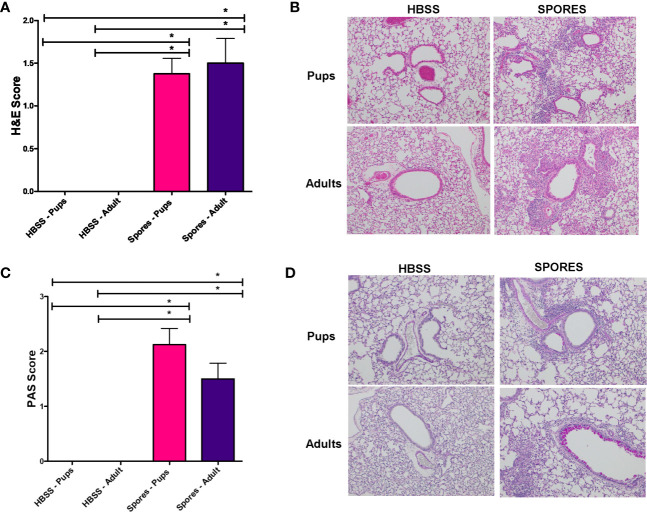
Histology analyses of adults and neonates. *p < 0.05, n = 8. **(A)** H&E scores. **(B)** Example H&E images. **(C)** PAS scores. **(D)** Example PAS images.

We further examined the tissue histology in adults and neonates. H&E scores (**Figure 6A**) and example figures (**Figure 6B**) were identical between adults and neonates, suggesting a similar level of lung tissue inflammation. MCM, measured by PAS staining, was significantly increased in both adults and neonates following exposure to spores (**Figures 6C, D**). But there was no difference between adults and neonates, suggesting that *Alternaria* spores induced the same level of lung remodeling in these two groups.

## Discussion

Mouse models of asthma are by far the most popular tool for asthma research. Nowadays, mice have become an ideal species for disease research because of the deep understanding of their genetics (Dietrich et al., 1996), the easy manipulation using gene-targeting technology (Elias et al., 2003) and the vast inventory of commercial mouse colonies. In general, mouse models of asthma have been developed by repeated sensitization with a number of established allergens such as ovalbumin (OVA) (Zosky et al., 2004), house dust mite (HDM) extract (Johnson et al., 2004), cockroach antigens (Lundy et al., 2003), and ragweed extracts (Fan and Jamal Mustafa, 2006). The OVA model or its many different variants is by far the most common model for asthma research. However, OVA is not a natural allergen for human asthma. Although other models indeed involve realistic human allergens (e.g. HMD, cockroach, or ragweed), they rely on artificial allergen-rich extracts that may not reflect real-life human exposures. Another limitation of these models is the difficulty to control batch effects of different preparations due to the lack of reliable internal standard of these complex mixtures.

Fungal exposure is universal from both indoor and outdoor environment and the prevalence of fungal sensitization in asthma is very high. The major form of fungal exposure is the inhalation of fungal spores (Pashley et al., 2012; Baxi et al., 2016). As spores can be grown *in vitro* and be titrated accurately, spore inhalation provides a unique model to study allergen sensitization in a natural condition, which none of the other models of allergen sensitization are able to mimic. However, filtrates (or filtrate extracts) are still main-stay allergens used in fungal asthma models, likely due to the commercially available materials at Greer Laboratory Inc. (Lenoir, NC). For four SAFS fungi (Denning et al., 2006), spore inhalation models of *Aspergillus* (Nayak et al., 2018) and *Cladosporium* (Denis et al., 2007) have been established. However, a previous attempt to establish an *Alternaria* spore model *via* a chronic inhalational exposure failed because of cachexia (Denis et al., 2007). In the present study, however, we did not observe any cachexia. Mice were given 10^5^ spores weekly that is in line with the dose of other spore inhalational model (Denis et al., 2007; Nayak et al., 2018), and they looked healthy and were also fertile. The cause of this discrepancy is not known, but we did use a different source of *Alternaria alternata* from the other study. A high level of TNFα production was speculated to be the cause of cachexia in the other study (Denis et al., 2007). It will be interesting to examine if our spores stimulate low or none TNFα production in the animal. Nonetheless, our mice remained healthy after a 6-week exposure and demonstrated several hallmark phenotypes of allergic asthma.

Fungal spores and filtrates represent very different components of *Alternaria*. The filtrates are the secretome of the fungus and has proteolytic activity and large amounts of allergens while the chitin encased spores are the transmissible, viable element. Respiratory exposure can occur with both. Despite comparable increases of AHR, tissue inflammation and MCM, the underlying immunological changes were different between the filtrate and spore model. Eosinophilia and antibody responses (both IgE and IgG1) were dominant in the filtrate model, while a mixed inflammatory profile with both neutrophils and eosinophils were present in the spore model. Neutrophilic inflammation has been a hallmark of fungal asthma, and neutrophils are a major defender against fungal exposure. Thus, the presence of a significant number of neutrophils in BAL may reflect the real physiological condition during fungal spore exposure. In contrast, the filtrate model represents a robust (perhaps overwhelming) T_H_2 allergic response that may be caused by a strong antigenicity of the filtrates. Indeed, the IgG1 level was also higher in the filtrate model. Another interesting finding is the lack of T_H_17 response in the filtrate model. Our finding in the chronic model is consistent with previous results from the acute filtrate exposure models. In one study, T_H_17 responses were found to be induced only by the co-exposure of both *Alternaria* filtrate and house dust mite extract, but not by each individual exposure (Snelgrove et al., 2014). In a separate study, IL-17A was found to be induced only in the absence of T_H_2 response but not in the normal condition (Valladao et al., 2016). Taken together, T_H_17 pathway appears to be missing in all filtrate exposure models. In contrast, the spore model demonstrated a robust T_H_17 response, which was also found to play an important role in antifungal defense (Werner et al., 2009; Werner et al., 2011) and in an aspergillus induced fungal asthma model (Murdock et al., 2012). Thus, although its robust allergic responses have been useful to dissect T2IR, the filtrate model may not be a good model for studying fungal asthma, since it is lack of T_H_17 component, a key player to drive steroid-resistant allergic airway inflammation that differentiates fungal asthma (McKinley et al., 2008). Nonetheless, the spore exposure model represents a novel and physiologically relevant model for the study of *Alternaria* induced fungal asthma.

As asthma starts from childhood, we have established a neonatal spore exposure model in this study. As compared to the adult model, the neonatal model demonstrated a similar level of allergy-related physiological responses (AHR, eosinophilia, MCM and IgE), supporting the notion that exposing to *Alternaria* spores causes asthma in both child and adult. Interestingly, spores induced significantly higher numbers of lymphocytes and levels of IgG1 in adults than in neonates. Neonates generally demonstrate weakened T_H_1 immune responses partially due to a biased T_H_2 response (Adkins et al., 2004; Levy, 2007), which could be an explanation for lower numbers of lymphocytes and IgG levels, but comparable levels of eosinophils and IgE, in neonates as compared to adults. *Alternaria* has been documented to cause severe fungal diseases (Pastor and Guarro, 2008) in addition to allergic asthma. Lower numbers of lymphocytes and low levels of IgG may be an indicator of weak anti-fungal immunity. It is unclear if infants are more prone to develop severe fungal disease when exposing to *Alternaria*, which warrants further study in the future. On the contrary, neonates who had an adult level of T_H_2 response may have an increased risk to develop allergic diseases such as asthma. Most strikingly, airway remodeling such as MCM was observed at comparable levels in both neonates and adults, raising the concern that neonatal exposure to fungal aeroallergen might cause a long-term sequalae.

In summary, we have successfully developed two novel models of fungal asthma by exposing either adult mice or neonates to live *Alternaria* spores. They will be valuable tools to dissect underlying pathogenic factors that contribute to adult or childhood asthma.

## Data Availability Statement

The raw data supporting the conclusions of this article will be made available by the authors, without undue reservation.

## Ethics Statement

The animal study was reviewed and approved by Institutional Animal Care and Use Committee (IACUC) at the University of Arizona.

## Author Contributions

MD and YC provided contributions in the conception or design of the work. RP, AC and DGB did the data collection. RP, AC, YL, QL, and DGB did data analysis and interpretation. YC and MD drafted the article. YC, MD, and BP contributed to the critical revision of the article. All authors contributed to the article and approved the submitted version.

## Funding

The study was supported partly by NIH grants AI149754, ES027013, AI39439, ES028889.

## Conflict of Interest

The authors declare that the research was conducted in the absence of any commercial or financial relationships that could be construed as a potential conflict of interest.

## Publisher’s Note

All claims expressed in this article are solely those of the authors and do not necessarily represent those of their affiliated organizations, or those of the publisher, the editors and the reviewers. Any product that may be evaluated in this article, or claim that may be made by its manufacturer, is not guaranteed or endorsed by the publisher.
